# Rapid development of a digital module during the Covid 19 pandemic in undergraduate medical education of pediatrics by teachers and students

**DOI:** 10.3205/zma001359

**Published:** 2020-12-03

**Authors:** Marie Mikuteit, Sandra Steffens, Lorenz Grigull, Lara Kühnle, Marianne Behrends, Ralf Schmidt, Urs Mücke

**Affiliations:** 1 Medizinische Hochschule Hannover, Hannover, Germany; 2 Universitätsklinikum Bonn, Bonn, Germany; 3 Gamespired - game-inspired thinking for your organization, Uplengen, Germany

## Abstract

**Objective: **During the early Covid 19 pandemic, undergraduate medical teaching of pediatric medicine had to be switched to online teaching at the Hanover Medical School (MHH). The aim was to develop an online module together with students.

**Methodology:** In a multi-stage process, a working group consisting of lecturers and students developed the concept and implemented it. Afterwards the online module was evaluated.

**Results:** The conceptualization process and the implementation of the module together with students can be represented as a modified PDCA cycle (Plan-Do-Check-Act). We showed that including students in the development of an online module is helpful in times of limited resources e.g. such as personnel and time.

**Conclusion:** The cooperation between students and lecturers is suitable for developing and implementing an online module in a short time. In the future, in addition to joint conceptualization phases, digital elements (e.g. preparatory webinars) for the module itself in attendance phases should be retained.

## Zusammenfassung

**Zielsetzung:** Im Rahmen der Covid-19-Pandemie musste die Lehre im Fach Kinderheilkunde im vierten Studienjahr an der Medizinischen Hochschule Hannover (MHH) auf reinen digitalen Unterricht umgestellt werden. Ziel war es, gemeinsam mit Studierenden das Modul zu digitalisieren.

**Methodik: **In einem mehrstufigen Prozess erarbeitete eine Arbeitsgruppe aus Dozierenden und Studierenden ein Konzept, um die bestehenden Inhalte digitalisiert anzubieten. Nach der Durchführung des Online-Moduls wurde dies evaluiert.

**Ergebnisse: **Der Konzeptionalisierungsprozess und die Umsetzung des Moduls gemeinsam mit Studierenden lassen sich als modifizierter PDCA-Zyklus (Plan-Do-Check-Act) darstellen. Wir zeigen Einsatzmöglichkeiten für Studierende zur verbesserten Gestaltung von Lehrveranstaltungen in Zeiten mit begrenzten Ressourcen wie Personal und Zeit auf. Die gezielte Einbindung von Studierenden wurde als vorteilhaft bewertet.

**Schlussfolgerung: **Die Zusammenarbeit von Studierenden und Dozierenden ist geeignet, um ein digitales Modul in kurzer Zeit zu entwickeln und durchzuführen. Zukünftig sollen neben gemeinsamen Konzeptionierungsphasen auch digitale Elemente (z.B. vorbereitende Webinare) für das Modul selbst in Präsenzphasen beibehalten werden.

## Introduction

Medical education is characterized by an ongoing exchange between fellow students, teachers and patients. Due to the Covid 19 pandemic, adjustments due to the hygiene regulations and other restrictions had to be made in a very short time. This presented the faculties with the challenge of offering practice- and case-oriented teaching in digital form [1]. Usually, students are not involved in the development process.

## Project description

Undergraduate medical education in pediatrics is located mainly in the fourth year of medical studies [2]. In addition to classical lectures and seminars (Problem Oriented Learning, POL), an interactive training for clinical decisions (KLE) was already established in a Learning Management System (LMS) ILIAS [https://elearning.mh-hannover.de/]. Within only six weeks an alternative concept for presence teaching in a digital learning environment had to be developed. The process of development can be transferred to other faculties.

## Results

The multi-step process was planned by two module managers (UM, LG) and executed by an interdisciplinary team of lecturers and students. The project phases were based on the PDCA cycle [3] and were divided into 

*planning:*

1. needs analysis,
2. actual state analysis,
3. conceptual design of a digital module structure;

*implementation:*

1. structure creation in the LMS, creation of new content;

*check:*

1. comparison with needs;

act:

1. implementation of the module. Subsequently, an evaluation and an internal university presentation followed.

In step 1 (informal group discussion) special needs were identified (see table 1 (Tab. 1)). In all PDCA phases, students could be specifically integrated. Four students were recruited at short notice.

**Figure 1:**
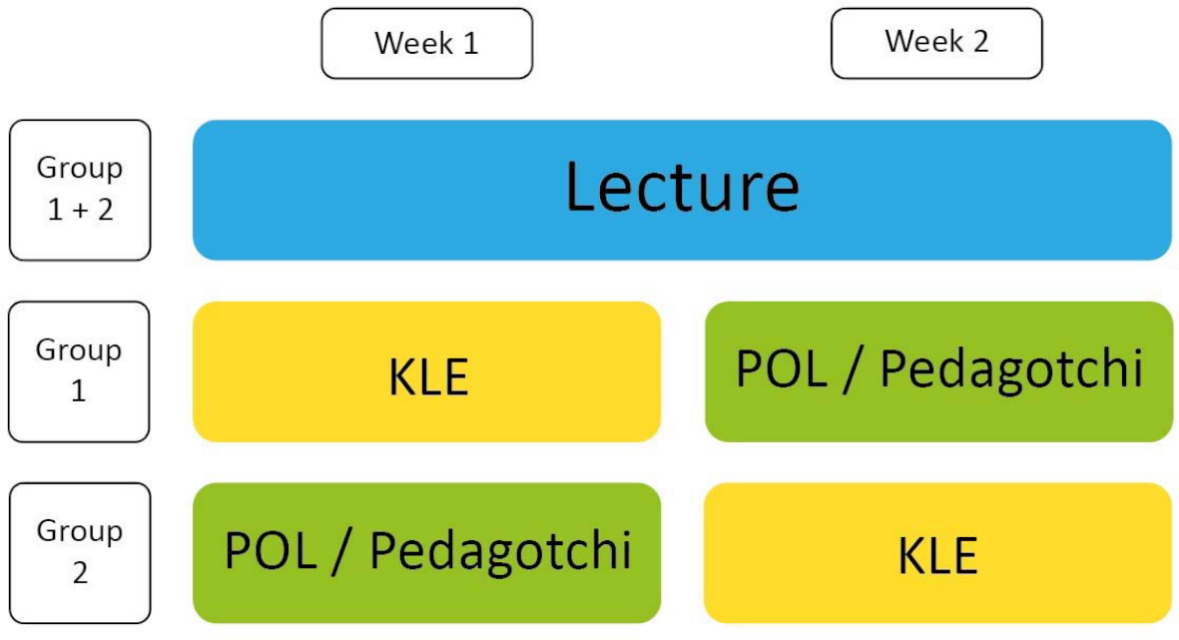
Structure of the digitalized module Pediatrics (POL=Problem-oriented learning, KLE=Training of clinical decisions)

The analysis showed that the existing LMS was suitable for the implementation of the contents. In addition, a teaching format for training clinical decisions had already been digitized. Addressing the students' concerns, learning content was offered in a daily structure. In addition to asynchronous self-study, synchronous webinars were organized in subgroups of 50 students, in which questions previously collected in forums and prioritized by the students were discussed (see figure 1 (Fig. 1) and table 2 (Tab. 2)). The number of semester hours was the same. A total of 102 students took part in the module. An evaluation (digital and anonymous) was answered by 51 students (12 men, 39 women). Videos as lecture substitutes were rated “very good” by 86%, even though only 61% rated the quality as good/very good. 37% of the students missed classical lectures in the lecture hall, 78% wanted seminars. The statement “My motivation to learn in the current module was comparable to or higher than previous modules with digital teaching” was agreed more or less by 89%. Overall, the students rated the digital module with 13 out of 15 points, which is no worse than the previous years' ratings for their analog module. The module was presented as a best practice example at an internal university event. Here, the cooperative design (students+teachers) was highlighted as an innovation worthy of consolidation. Despite different starting situations, the development of a digital module could succeed in cooperation with students from other faculties. 

## Discussion

It was not until the pressure for digitization that a stronger integration of learning content into the existing LMS was achieved. The students' good knowledge of the LMS was a strategic advantage for the necessary work [4]. Already digitally based formats (e.g. Pedagotchi [5] and KLE) proved to be easily scalable to larger groups. Blended learning formats, which could be integrated at a low level, can be used to train clinical skills that go beyond mere knowledge transfer [6].

The conceptualization together with students proved to be a suitable way to digitize traditional formats according to the needs of the students. This concept was also included in the students’ position paper on digitization in university education [7] and can be transferred to other faculties. The evaluation of the module showed a high acceptance of digital learning units despite technical improvements, which is generally often the case, regardless of the content [8]. One limitation is the lack of comparison with another cohort.

Nevertheless, learning with patients – in contrast to digital cases – leads to a better awareness of the problem [9]. Therefore, a hybrid module with webinars and face-to-face seminars with patients is to be developed in the future. Both new developments from the pandemic period as well as impulses from students can be included. 

## Conclusion

Corona-related limitations have given a strong impetus to collaboration and digitization. The integration of students can be implemented at a low-threshold and has a positive effect on the further development of existing formats. The fully digitized content now offers the possibility of a flexible response to changing challenges in teaching.

## Competing interests

The authors declare that they have no competing interests.

## Figures and Tables

**Table 1:**
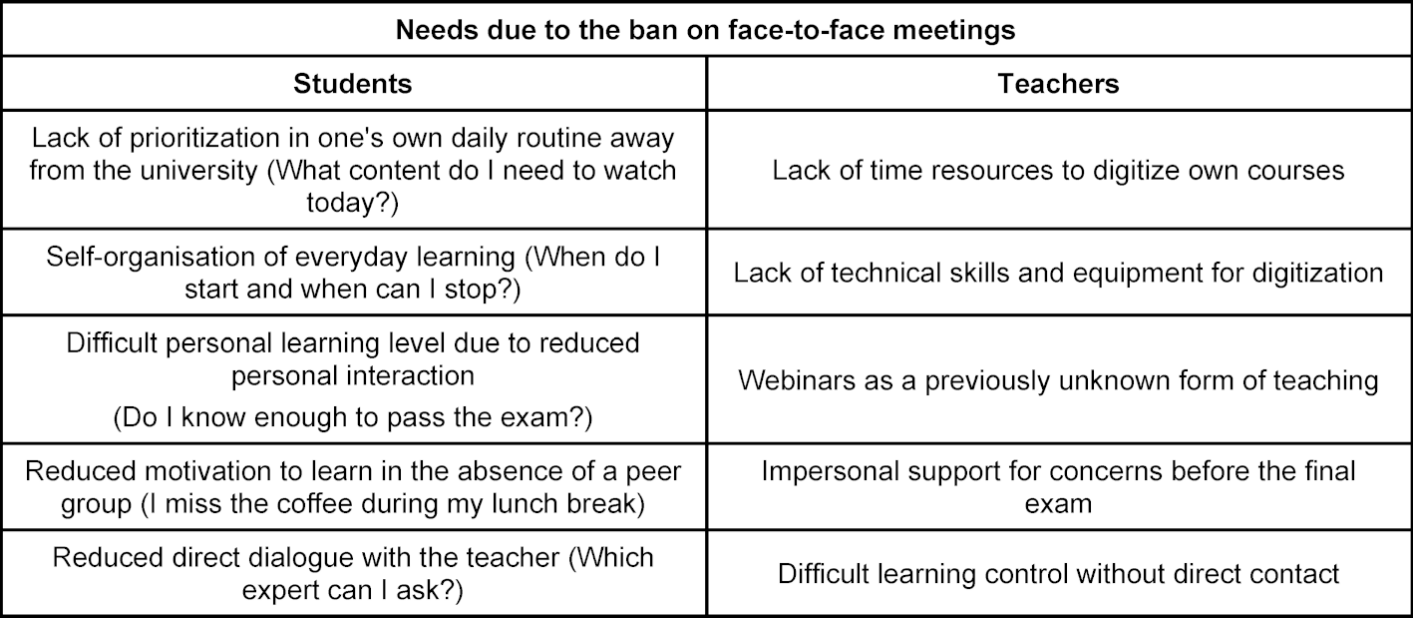
Needs due to the ban on face-to-face meetings (results of a group discussion)

**Table 2:**
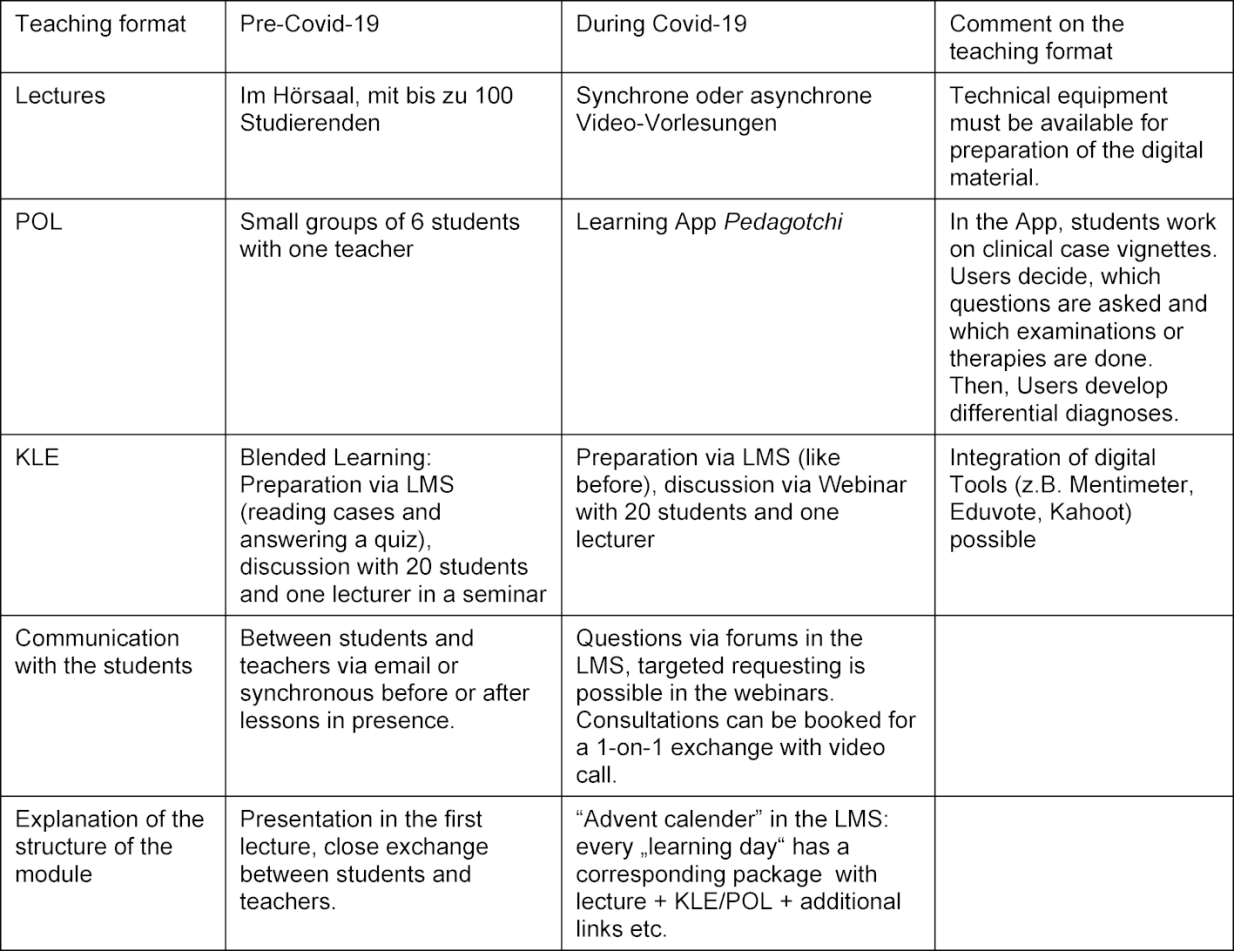
Teaching formats before and during the covid-19 pandemic

## References

[1] Arandjelovic A, Arandjelovic K, Dwyer K, Shaw C (2020). COVID-19: Considerations for Medical Education during a Pandemic. MedEdPublish.

[2] Medizinische Hochschule Hannover (2019). Curriculum des Moduls Kinderheilkunde und Jugendmedizin der MHH.

[3] Behrend R, Mette M, Partecke M, Reichel K, Wershofen B (2019). Heterogeneous learning cultures in interprofessional education: a teacher training. GMS J Med Educ.

[4] Sandars J, Correia R, Dankbaar M, de Jong P, Goh PS, Hege I, Masters K, Oh SY, Patel R, Premkumar K, Webb A, Pusic M (2020). Twelve tips for rapidly migrating to online learning during the COVID-19 pandemic. MedEdPublish.

[5] Schmidt R, Grigull L (2018). Pedagotchi: Entwicklung einer neuartigen Lernanwendung für die Pädiatrie. Monatsschr Kinderheilkd.

[6] Rowe M, Frantz J, Bozalek V (2012). The role of blended learning in the clinical education of healthcare students: A systematic review. Med Teach.

[7] Baumann J, Böckel A, Denker F, Gross P, Kern E, Lamprecht M, Reimann J, Rensinghoff B, Sari Z, Schopf E, Wächtler E, Meyer H, Rampelt F, Röwert R (2019). Der Digital Turn aus Studierendenperspektive. Studentisches Thesenpapier zur Digitalisierung in der Hochschulbildung. Diskussionspapier Nr.7.

[8] Kühl SJ, Toberer M, Keis O, Tolks D, Fischer MR, Kühl M (2017). Concept and benefits of the Inverted Classroom method for a competency-based biochemistry course in the pre-clinical stage of a human medicine course of studies. GMS J Med Educ.

[9] Li J, Li QL, Li J, Chen ML, Xie HF, Li YP, Chen X (2013). Comparison of three problem-based learning conditions (real patients, digital and paper) with lecture-based learning in a dermatology course: a prospective randomized study from China. Med Teach.

